# The Increased Expression of Matrix Metalloproteinases Associated with Elastin Degradation and Fibrosis of the Ligamentum Flavum in Patients with Lumbar Spinal Stenosis

**DOI:** 10.4055/cios.2009.1.2.81

**Published:** 2009-05-27

**Authors:** Jong-Beom Park, Chae-Gwan Kong, Kyung-Hwan Suhl, Eun-Deok Chang, K. Daniel Riew

**Affiliations:** Department of Orthopaedic Surgery, The Catholic University of Korea School of Medicine, Uijeongbu, Korea.; * Department of Pathology, The Catholic University of Korea School of Medicine, Uijeongbu, Korea.; † Department of Orthopaedic Surgery, Washington University School of Medicine, St. Louis, USA.

**Keywords:** Spinal stenosis, Ligamentum flavum, Elastin degradation, Fibrosis, Matrix metalloproteinases

## Abstract

**Background:**

One of the characteristics of spinal stenosis is elastin degradation and fibrosis of the extracellular matrix of the ligamentum flavum. However, there have been no investigations to determine which biochemical factors cause these histologic changes. So we performed the current study to investigate the hypothesis that matrix metalloproteinases (MMPs), which possess the ability to cause extracellular matrix remodeling, may play a role as a mediator for this malady in the ligamentum flavum.

**Methods:**

The ligamentum flavum specimens were surgically obtained from thirty patients with spinal stenosis, as well as from 30 control patients with a disc herniation. The extents of ligamentum flavum elastin degradation and fibrosis were graded (grade 0-4) with performing hematoxylin-eosin staining and Masson's trichrome staining, respectively. The localization of MMP-2 (gelatinase), MMP-3 (stromelysin) and MMP-13 (collagenase) within the ligamentum flavum tissue was determined by immunohistochemistry. The expressions of the active forms of MMP-2, MMP-3 and MMP-13 were determined by western blot analysis, and the blots were quantified using an imaging densitometer. The histologic and biochemical results were compared between the two conditions.

**Results:**

Elastin degradation and fibrosis of the ligamentum flavum were significantly more severe in the spinal stenosis samples than that in the disc herniation samples (3.14 ± 0.50 vs. 0.55 ± 0.60, *p* < 0.001; 3.10 ± 0.57 vs. 0.76 ± 0.52, *p* < 0.001, respectively). The expressions of the active form of MMPs were identified in all the ligamentum flavums of the spinal stenosis and disc herniation patients. The expressions of active MMP-2 and MMP-13 were significantly higher in the spinal stenosis samples than that in the disc herniation samples (both *p* < 0.05). The expression of active MMP-3 was slightly higher in the spinal stenosis samples than that in the disc herniation samples, but the difference was not statistically significant (*p* = 0.131). MMP-2, -3, and -13 were positively stained on the ligamentum flavum fibroblasts.

**Conclusions:**

The current results suggest that the increased expression of active MMPs by the ligamentum flavum fibroblasts might be related to the elastin degradation and fibrosis of the ligamentum flavum in the patients who suffer with lumbar spinal stenosis.

The ligamentum flavum is composed of elastin and collagen fibers in a 2:1 ratio. The elastin fibers provide elasticity to the ligament, while the collagen fibers provide stiffness and stability. With age, the elastin to collagen ratio decreases, and this causes decreased elasticity and increased stiffness or fibrosis. As these age-related degenerative changes progress, the ligamentum flavum tissues ultimately become disorganized, which contributes to the development of spinal stenosis. Previous studies have mainly investigated the histologic changes in the ligamentum flavum of spinal stenosis patients and they reported that there was degradation of elastin fibers, with an increase in collagen fibers, fibrosis and calcification within the hypertrophied ligamentum flavum tissues.^1^-5) However, to the best our knowledge, there has been no study that has investigated the biochemical factors associated with these histologic changes.

Matrix metalloproteinases (MMPs) are a family of over 20 zinc-dependent enzymes that degrade all kinds of extracellular matrix components such as elastin, collagen and proteoglycans.^6^-8) MMPs are responsible for the remodeling of connective tissues under normal physiologic and pathologic conditions. According to their substrate specificity, the classic MMPs fall into three main groups: collagenases (MMP-1, 8, -13), gelatinases (MMP-2, -9), and stromelysins (MMP-3, -7, -10). The MMPs are constitutively expressed in various types of cells such as parenchymal, connective tissue and inflammatory cells. The activity of MMPs is strictly regulated at the level of synthesis and activation and by the interaction with endogenous inhibitors under normal physiologic conditions. Increased MMP activity can result in several pathologic conditions such as arthritis, aneurysm, atherosclerosis, mid-dermal elastolysis, Marfan syndrome and intervertebral disc degeneration and herniation.^9^-15) These findings suggest that MMPs might also act as a mediator of elastin degradation and fibrosis of the ligamentum flavum, which occurs in spinal stenosis.

The purpose of the current study is to determine (1) whether the ligamentum flavum of spinal stenosis patients has more severe elastin degradation and fibrosis compared to that of patients with disc herniation and (2) whether there is an increased expression of MMPs in the ligamentum flavum of spinal stenosis patients compared to that of disc herniation patients and (3) the source of the MMPs within the ligamentum flavum tissues.

## METHODS

Thirty ligamentum flavum specimens were obtained from 30 patients who had undergone decompressive laminectomy for neurogenic claudication, which was due to degenerative lumbar spinal stenosis. The patients suffering with degenerative spondylolisthesis were not included in this study. We tried to obtain the entire layer of the central portion of the ligamentum flavum, and we removed all the epidural fat from the ligamentum flavum specimens. Half of each specimen was fixed in 4% neutral formalin, decalcified with 20% ethylenediaminetetraacetic acid (EDTA) for four to six weeks and then embedded in paraffin for histologic and immunohistochemical analyses; the other half was kept in a freezer at -0℃ for subsequent western blot analysis. We randomly selected thirty gender-matched control patients with lumbar disc herniation from a group of 234 patients who were operatively managed for that disorder.

### Histologic Analysis for Elastin Degradation and Fibrosis of the Ligamentum Flavum

Two consecutive sections (4-µm thick) were cut on a microtome and these were stained by Masson's trichrome and hematoxylin-eosin stains, respectively. Masson's trichrome stain was used to evaluate the degree of fibrosis and hematoxylin-eosin stain was used to evaluate the elastin degradation (loss, fragmentation and disorganization). Histologic analysis was independently performed by two pathologists on 10 randomly selected, high power fields (× 400) of each sample. The average of the 20 grades of fibrosis and elastin degradation was used as the final grade, respectively.

#### Masson's trichrome stain

**Fig. 1:**
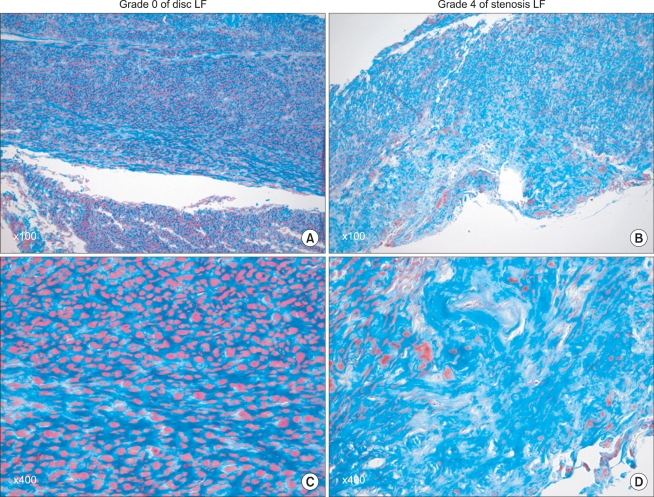
Grading of the ligamentum flavum fibrosis by Masson's trichrome staining. In the grade 0 samples (A and C), all the area of the ligamentum flavum was stained a pink color, indicating a normal, non-fibrotic state. However, in the grade 4 samples (B and D), most of the area was stained a blue color, indicating that most of the area was fibrotic.

The severity of ligamentum flavum fibrosis was graded according to the guidelines presented by Sairyo et al.16) Grade 0 indicates normal tissue showing no fibrotic region, grade 1 indicates fibrosis at < 25% of the entire area, grade 2 indicates between 25% and 50% fibrosis of the entire area, grade 3 indicates between 50% and 75% fibrosis and grade 4 indicates > 75% fibrosis (Fig. 1).

#### Hematoxylin-eosin stain

**Fig. 2:**
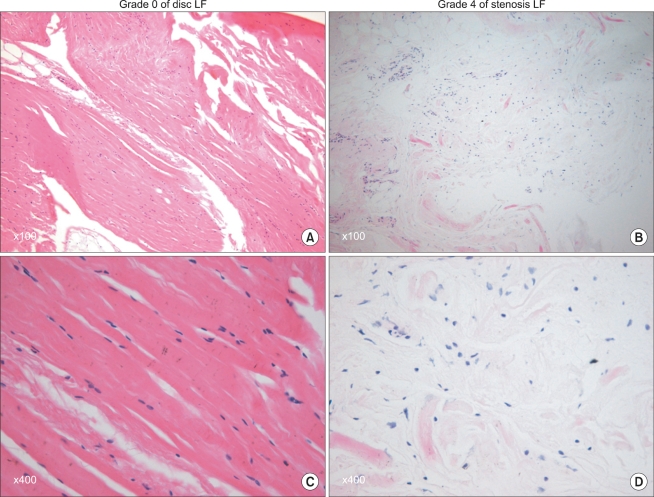
Grading of the ligamentum flavum elastin degradation by hematoxylin-eosin staining. In the grade 0 samples (A and C), rich normal elastin fibers were organized in a strictly parallel order. However, in the grade 4 samples (B and D), the residual scanty elastin fivers were fragmented and disorderly.

The degree of ligamentum flavum elastin degradation was also graded using the same scoring system as the fibrosis score. Grade 0 indicates normal tissue showing no elastin degradation region, grade 1 indicates elastin degradation at < 25% of the entire area, grade 2 indicates between 25% and 50% elastin degradation, grade 3, between 50% and 75% and grade 4 > 75% elastin degradation (Fig. 2).

### Immunohistochemical Analysis for the Localization of MMP-2, MMP-3 and MMP-13

Three consecutive sections (4-µm thick) were cut on a micro-tome and these were deparaffinized in xylene and then they were rehydrated in a graded series of alcohol solutions. To determine the expressions of MMP-2, MMP-3 and MMP-13, the avidin-biotin-peroxidase complex method and a Histostain™-plus SP kit (Zymed Laboratory Inc., South San Francisco, CA, USA) were used with following the manufacturer's instructions. Purified rabbit polyclonal antibody specific to MMP-2 (Thermo Scientific, San Diego, CA, USA), purified goat polyclonal antibody specific to MMP-3 (Santa Cruz Biotech, Santa Cruz, CA, USA) and purified rabbit polyclonal antibody to MMP-13 (Thermo Scientific) were used for this study at an optimum dilution recommended by the manufacturers. The positive controls were also stained according to the manufacturer's recommendation.

### Western Blot Analysis for the Expressions of Active MMP-2, MMP-3 and MMP-13

100 mg of ligamentum flavum tissue was homogenized with fetal bovine serum at 3000 rpm (Tissue Tearor/Biospec products/model 985-370, Bio-Spec Products, Racine, WI, USA) and then this was lysed in lysis buffer. The supernatant was obtained following centrifugation at 1500 rpm at 4℃ for 30 minutes. Quantification of protein was performed according to Bradford's method with using a protein assay kit (Bio-Rad/Cat No. 500-0006, Pierce Chemical Company, Rockford, IL, USA) and the reading was done at 595 nm by employing a spectrophotometer (Ultrospec 3000, Pharmacia Biotech, Cambridge, United Kingdom). For all of the analyses for each sample, 30 µg of protein were loaded onto a 12% sodium dodecyl sulfate (SDS)-polyacrylamide gel. The protein concentration of the lysed ligamentum flavum tissue was determined with using a bicinchoninic acid (BCA) protein assay reagent kit (Bio-Rad 500-0006, Pierce Chemical Company, Rockford, IL, USA). After electrophoresis, the proteins were transferred to a polyvinylidene difluoride (PVDF) membrane (Millipore, St. Quentin, Yvelines, France) for two hours at 150 V with using a transfer buffer. After blocking the nonspecific binding sites overnight with 5% non-fat milk phosphate buffer stock-Tween 20 (TPBS), the membranes were incubated for two hours at room temperature with purified rabbit polyclonal antibody specific to MMP-2, purified goat polyclonal antibody specific to MMP-3 and purified rabbit polyclonal antibody to MMP-13. Antibody labeling was identified using HRP-conjugated secondary antibodies (Amersham Life Sciences, Arlington Heights, IL, USA), and the results were visualized using enhanced chemiluminescence (ECL) (Amersham Life Sciences, Piscataway, NJ, USA). β-actin was used as an internal control for protein loading. The blots were quantified using an Imaging Densitometer GF670 and molecular analysis software (Bio-Rad, SAFC Bioscience, Sigma-Aldrich, USA) three times for each sample and the average of the three densities was used as the final density. The density is presented as the mean ± standard deviation (arbitrary units).

### Statistical Analysis

The Mann-Whitney U test was used to assess the difference in the degree of ligamentum flavum elastin degradation and fibrosis and the density of active MMP-2, -3, and -13 between the spinal stenosis samples and the disc herniation samples. A *p* value of less than 0.05 was considered statistically significant.

## RESULTS

### Demographic Data

Among the 30 patients with spinal stenosis, 23 were women and 7 were men. The mean age of the patients at the time of surgery was 63.1 years (range, 51 to 77 years), and the mean duration of symptoms between onset and operation was 13.1 weeks (range, 4 to 29 weeks). The mean thickness of the ligamentum flavum, as measured on the T1-weighted axial image of the facet joint level of the lesion, was 5.4 mm (range, 4.2 to 7.3 mm). Of the 30 ligamentum flavum specimens, 21 were obtained from the L4-L5 level, seven from the L5-S1 level and two from the L3-4 level. The mean age of the patients with disc herniation was 31.1 years (range, 19 to 41 years). The thickness of the ligamentum flavum, as measured on the T1-weighted axial image of the facet joint level of the lesion, was 2.3 mm (range, 1.5 to 3.2 mm).

### Elastin Degradation and Fibrosis of the Ligamentum Flavum

The mean grade of elastin degradation of the ligamentum flavum was significantly higher in the stenosis samples than that in the disc herniation samples (3.14 ± 0.50 vs. 0.55 ± 0.60, respectively, *p* < 0.001). The mean grade of ligamentum flavum fibrosis was also significantly higher in the stenosis specimens than that in the disc herniation samples (3.10 ± 0.57 vs. 0.76 ± 0.52, respectively, *p* < 0.001).

### Localization of MMP-2, MMP-3 and MMP-13

**Fig. 3:**
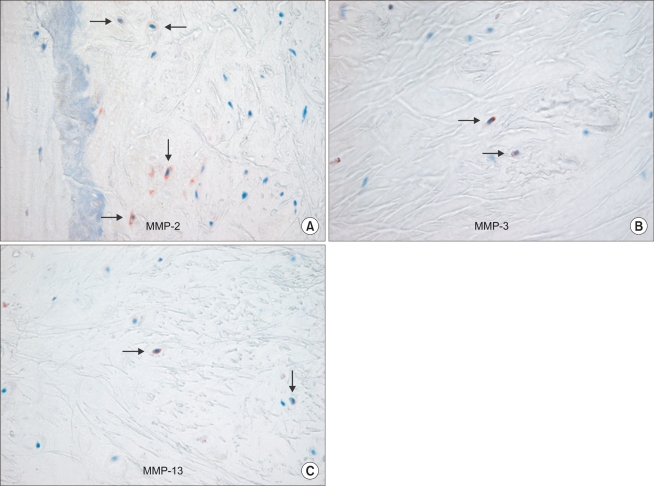
Immunohistochemical analysis demonstrates that MMP-2 (A), MMP-3 (B), and MMP-13 (C) were positively stained on the ligamentum flavum fibroblasts of the patients with spinal stenosis (original magnifi cation, × 400).

Immunohistochemical analysis demonstrated that MMP-2, MMP-3, and MMP-13 were positively stained on the ligamentum flavum fibroblasts of the patients with spinal stenosis and disc herniation, respectively (Fig. 3).

### Expressions of MMP-2, MMP-3, and MMP-13

**Fig. 4:**
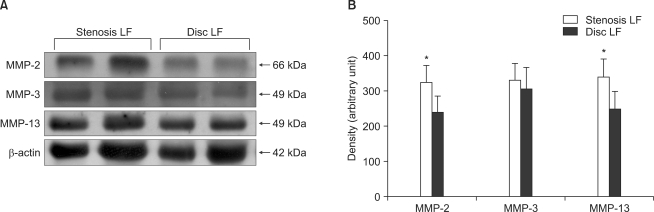
Western blot analysis (A) demonstrated the expressions of active MMP-2, MMP-3 and MMP-13 in the ligamentum flavum specimens of the spinal stenosis and disc herniation patients, and the blots were quantified using an Imaging Densitometer. (B) Density is presented as the mean ± standard deviation (arbitrary units). The mean density of active MMP-2 and MMP-13 was statistically higher in the spinal stenosis samples than that in the disc herniation samples (both *p* < 0.05). Although the mean density of active MMP-3 was slightly higher in the spinal stenosis samples than that in the disc herniation samples, the difference was not statistically significant (*p* = 0.131). ^*^ *p* < 0.05.

Western blot analysis demonstrated the expressions of active MMP-2, MMP-3, and MMP-13 in the ligamentum flavum specimens of the spinal stenosis patients and the disc herniation patients (Fig. 4A). The mean density (arbitrary units) of active MMP-2 and MMP-13 was statistically higher in the spinal stenosis specimens than that in the disc herniation specimens (324.75 ± 63.66 vs. 238.00 ± 76.05, respectively, *p* < 0.05; 340.00 ± 48.63 vs. 248.50 ± 52.09, respectively, *p* < 0.05). Although the mean density (arbitrary units) of active MMP-3 was slightly higher in the spinal stenosis specimens than that in the disc herniation specimens, the difference was not statistically significant (329.24 ± 65.91 vs. 305.24 ± 71.35, respectively, *p* = 0.131) (Fig. 4B).

## DISCUSSION

The initial studies on the ligamentum flavum in patients with spinal stenosis revealed profound histologic changes, including hypertrophy, fibrosis and loss of elastin. Sairyo et al.16) examined the ligamentum flavum specimens from patients with spinal stenosis or discogenic back pain and they were the first to report a positive relationship between the thickness of the ligamentum flavum and the loss of elasticity and fibrosis. However, one criticism of that primary study is that the specimens from the two conditions were analyzed as one group that was differentiated only by the degree of thickness of the ligamentum flavum. We sought to further characterize these findings regarding the ligamentum by comparing the degree of elastin degradation and fibrosis in spinal stenosis patients to that of a control group of disc herniation patients.

In addition, we sought to determine which bio chemical factors might be responsible for these changes. Recent reports have suggested that increased concentrations of transforming growth factor-beta 1 (TGF-β1) or tissue inhibitors of metalloproteinases (TIMPs) might be associated with ligamentum flavum hypertrophy in patients with spinal stenosis.^17^-20) It is well known that MMPs can degrade elastin and collagen components within the various connective tissues of the body.^6^-8) Based on this, we hypothesized that MMPs might also play a role on the histologic changes that occur in the ligamentum flavum of spinal stenosis patients. We chose to examine three MMPs: MMP-2 (gelatinase), which most prominently possesses the ability to degrade elastin; MMP-3 (stromelysin), which digests various components of the extracellular matrix like proteoglycans, fibronectin, collagen, laminin, gelatin and elastin and they can activate other MMPs, and MMP-13 (collagenase), which is known to be involved in collagen degradation. To the best of our knowledge, this is the first study to compare the degree of ligamentum flavum elastin degradation and fibrosis in spinal stenosis patients as compared to that in more normal controls, and to further characterize the possible biochemical etiology of those changes.

We found that the mean grades of elastin degradation and fibrosis of the ligamentum flavum were statistically higher in the spinal stenosis samples than that in the disc herniation samples. The thickness of the ligamentum flavum tissues was also statistically higher in the spinal stenosis samples than that in the disc herniation samples. In addition, we found that the expression of MMP-2 was statistically higher in the patients with spinal stenosis than it was in those patients with disc herniation (*p* < 0.05). Considering the well-known ability of MMP-2 to degrade elastin, its high expression in the ligamentum flavum of spinal stenosis patients suggests that it may be, at least partially, responsible for the severe loss and disorganization of the elastin fibers observed in that pathological condition. We also found that there was a higher expression of MMP-13 in the spinal stenosis samples compared with that of the disc herniation samples (*p* < 0.05). Considering the ability of MMP-13 to degrade collagen, this result might seem contradictory for a condition where the collagen hypertrophies. However, the increased collagen fibers in the ligamentum flavum of the spinal stenosis samples were due to an increased deposition of abnormally disordered collagen fibers, and not normal collagen fibers. Previous studies have suggested that TGF-β1 synthesized by the ligamentum flavum fibroblasts increases collagen synthesis, resulting in hypertrophy.^17^-19) Therefore, it is suggested that increased fibrosis of the ligamentum flavum in spinal stenosis patients is a net result of a series of synthesis and destruction of the collagen fibers by TGF-β1 and MMP-13. The MMP-3 expression was slightly higher in the patients with spinal stenosis than that in the disc herniation patients, but the difference was not statistically significant (*p* = 0.131). It has been reported that MMP-3 plays a central role in early matrix destruction, whereas it is down-regulated in the late stages of matrix destruction.^21^,22) Considering that the expression pattern of MMP-3 depends on the disease stage, it is not unexpected that the expression of MMP-3 was not significantly higher in the spinal stenosis ligamentum flavum samples with their elastin degradation, and fibrosis is thought to be a chronic or late-stage finding of spinal stenosis. Finally, we detected MMP-2, MMP-3 and MMP-13 immunoreactivities in the cytoplasm of the fibroblasts from the ligamentum flavum tissues of both conditions, indicating that these MMPs were synthesized in these cells.

The activity of MMPs can be affected by several factors, and especially TIMPs. TIMPs bind strongly but noncovalently to activated MMPs. TIMPs are co-expressed with MMPs and they contribute to the regulation of their activity so that increases in the TIMP levels reduce MMP activity. However, contradictory results regarding the delicate balance between MMPs and TIMPs have been reported in several pathologic conditions such as abdominal aortic aneurysms, chronic liver injury and osteoarthritis.^23^-26) A recent study by Park et al.20) suggested that the increased expression of TIMPs in ligamentum flavum fibroblasts is associated with its fibrosis and hypertrophy of the ligamentum flavum in patients suffering with spinal stenosis. On the basis of that study and the present findings, the combined increased expressions of both MMPs and TIMPs suggest that the possibility of a vicious cycle whereby the presence of denatured extracellular matrix components could lead to increased MMP production, which in turn could generate more denatured extracellular matrix components. The profound destruction of the extracellular matrix components in spinal stenosis patients may stimulate a further increased expression of TIMPs. Further understanding the expression levels of MMPs and TIMPs for the various degrees of elastin degradation, fibrosis, and hypertrophy of the ligamentum flavum is necessary to define the exact relationship between these factors and the role that they play during the various stages of spinal stenosis.

As with any study, our study had a few shortcomings. While we found increases of MMP-2 and MMP-13 in the spinal stenosis samples, this does not necessarily mean that these factors are either solely or even partially responsible for the histologic changes associated with the clinical condition. The increases may be associated findings, but not necessarily interrelated findings. Nevertheless, given the known activities and roles that these enzymes play, we believe that it is reasonable to postulate that there might be a cause and effect relationship present. Secondly, we used control specimens from patients with disc herniations and whose average age was 31.1 years, which was significantly younger than that for the specimens from the patients with spinal stenosis. It would have been ideal to have two different controls: age and gender matched specimens from patients without stenosis and gender matched specimens from late-teenagers. The former would eliminate age as an independent variable, since not all older patients develop spinal stenosis. It may be that, much like atherosclerosis, a genetic predisposition alters the biochemical factors that contribute to the pathologic condition. Specimens from late-teenagers who are without any degenerative changes would allow us to determine the true extent of the perturbations that occur in spinal stenosis. Unfortunately, both of these ideal controls rarely undergo surgical treatment, making it nearly impossible to gather enough samples in a timely fashion. Thus, we finally settled on our present controls, which although admittedly they are not ideal, we believe they are reasonable and adequate to address the questions that we have posed.

In conclusion, we found an increased expression of active MMPs in the ligamentum flavum fibroblasts of spinal stenosis patients as compared with those of disc herniation patients. In addition, there was more severe elastin degradation and fibrosis of the ligamentum flavum in the spinal stenosis patients than that in the disc herniation patients. These results suggest that an increased expression of active MMPs by the ligamentum flavum fibroblasts might be related to the elastin degradation and fibrosis of the ligamentum flavum in the patients who suffer with lumbar spinal stenosis.

## References

[1] Schrader PK, Grob D, Rahn BA, Cordey J, Dvorak J (1999). Histology of the ligamentum flavum in patients with degenerative lumbar spinal stenosis. Eur Spine J.

[2] Yoshida M, Shima K, Taniguchi Y, Tamaki T, Tanaka T (1992). Hypertrophied ligamentum flavum in lumbar spinal canal stenosis: pathogenesis and morphologic and immunohistochemical observation. Spine.

[3] Okuda T, Baba I, Fujimoto Y (2004). The pathology of ligamentum flavum in degenerative lumbar disease. Spine.

[4] Postacchini F, Gumina S, Cinotti G, Perugia D, DeMartino C (1994). Ligamenta flava in lumbar disc herniation and spinal stenosis: light and electron microscopic morphology. Spine.

[5] Yahia H, Drouin G, Maurais G, Garzon S, Rivard CH (1989). Degeneration of the human lumbar spine ligaments: an ultrastructural study. Pathol Res Pract.

[6] Nagase H, Woessner JF (1999). Matrix metalloproteinases. J Biol Chem.

[7] Johnson LL, Dyer R, Hupe DJ (1998). Matrix metalloproteinases. Curr Opin Chem Biol.

[8] Woessner JF (1991). Matrix metalloproteinases and their inhibitors in connective tissue remodeling. FASEB J.

[9] Dean DD, Martel-Pelletier J, Pelletier JP, Howell DS, Woessner JF (1989). Evidence for metalloproteinase and metalloproteinase inhibitor imbalance in human osteoarthritic cartilage. J Clin Invest.

[10] Basalyga DM, Simionescu DT, Xiong W, Baxter BT, Starcher BC, Vyavahare NR (2004). Elastin degradation and calcification in an abdominal aorta injury model: role of matrix metalloproteinases. Circulation.

[11] Robert L, Robert AM, Jacotot B (1998). Elastin-elastase-atherosclerosis revisited. Atherosclerosis.

[12] Patroi I, Annessi G, Girolomoni G (2003). Mid-dermal elastolysis: a clinical, histologic, and immunohistochemical study of 11 patients. J Am Acad Dermatol.

[13] Chung AW, Au Yeung K, Sandor GG, Judge DP, Dietz HC, van Breemen C (2007). Loss of elastic fiber integrity and reduction of vascular smooth muscle contraction resulting from the upregulated activities of matrix metalloproteinase-2 and -9 in the thoracic aortic aneurysm in Marfan syndrome. Circ Res.

[14] Weiler C, Nerlich AG, Zipperer J, Bachmeier BE, Boos N (2002). 2002 SSE Award Competition in Basic Science: expression of major matrix metalloproteinases is associated with intervertebral disc degradation and resorption. Eur Spine J.

[15] Goupille P, Jayson MI, Valat JP, Freemont AJ (1998). Matrix metalloproteinases: the clue to intervertebral disc degeneration?. Spine.

[16] Sairyo K, Biyani A, Goel VK (2007). Lumbar ligamentum flavum hypertrophy is due to accumulation of inflammation-related scar tissue. Spine.

[17] Park JB, Chang H, Lee JK (2001). Quantitative analysis of transforming growth factor-beta 1 in ligamentum flavum of lumbar spinal stenosis and disc herniation. Spine.

[18] Nakatani T, Marui T, Hitora T, Doita M, Nishida K, Kurosaka M (2002). Mechanical stretching force promotes collagen synthesis by cultured cells from human ligamentum flavum via transforming growth factor-beta1. J Orthop Res.

[19] Fukuyama S, Nakamura T, Ikeda T, Takagi K (1995). The effect of mechanical stress on hypertrophy of the lumbar ligamentum flavum. J Spinal Disord.

[20] Park JB, Lee JK, Park SJ, Riew KD (2005). Hypertrophy of ligamentum flavum in lumbar spinal stenosis associated with increased proteinase inhibitor concentration. J Bone Joint Surg Am.

[21] Aigner T, Zien A, Gehrsitz A, Gebhard PM, McKenna L (2001). Anabolic and catabolic gene expression pattern analysis in normal versus osteoarthritic cartilage using complementary DNA-array technology. Arthritis Rheum.

[22] Cawston T, Billington C, Cleaver C (1999). The regulation of MMPs and TIMPs in cartilage turnover. Ann N Y Acad Sci.

[23] Ishiguro N, Ito T, Ito H (1999). Relationship of matrix metalloproteinases and their inhibitors to cartilage proteoglycan and collagen turnover: analyses of synovial fluid from patients with osteoarthritis. Arthritis Rheum.

[24] Booms P, Pregla R, Ney A (2005). RGD-containing fibrillin-1 fragments upregulate matrix metalloproteinase expression in cell culture: a potential factor in the pathogenesis of the Marfan syndrome. Hum Genet.

[25] Knittel T, Mehde M, Grundmann A, Saile B, Scharf JG, Ramadori G (2000). Expression of matrix metalloproteinases and their inhibitors during hepatic tissue repair in the rat. Histochem Cell Biol.

[26] Yoshihara Y, Nakamura H, Obata K (2000). Matrix metalloproteinases and tissue inhibitors of metalloproteinases in synovial fluids from patients with rheumatoid arthritis or osteoarthritis. Ann Rheum Dis.

